# Prediction and prevention of preeclampsia by physicians in Brazil: An original study

**DOI:** 10.3389/fgwh.2022.983131

**Published:** 2022-10-19

**Authors:** Edson Vieira da Cunha Filho, Tamara Cristina Gomes Ferraz Rodrigues, Valeria Cristina Sandrim, Eduardo Carvalho de Arruda Veiga, Ricardo Carvalho Cavalli

**Affiliations:** ^1^Hospital Moinhos de Vento, Porto Alegre, RS, Brazil; ^2^Department of Gynecology and Obstetrics, Ribeirão Preto Medical School, University of São Paulo, São Paulo, Brazil; ^3^Department of Pharmacology and Biophysics, Institute of Biosciences, São Paulo State University (Unesp), São Paulo, Brazil

**Keywords:** preeclampsia, prediction, prevention, aspirin, calcium, uterine artery Doppler

## Abstract

**Background:**

Considering the worldwide importance of preeclampsia, especially in Brazil, the screening of pregnant women at greater risk of developing the disease and the application of preventive measures are essential. This study aimed to assess the medical performance in this context in Brazil.

**Methods:**

A survey was developed to quantify the number of physicians who prescribe acetylsalicylic acid (ASA) and/or calcium for preeclampsia prevention. The survey was sent to all Brazilian obstetricians affiliated to the Brazilian Federation of OBGYN by email and WhatsApp. The survey remained opened for 6 months and included questions about the use of ASA and calcium, as well as about the use of a complementary test to predict preeclampsia.

**Results:**

The sample consisted of 360 responding physicians and 100% coverage of responses from physicians from the five different regions of Brazil was obtained. The vast majority of respondents (94.72%) prescribe ASA to prevent preeclampsia, with 80.3% prescribing a dose of 100 mg/day. Calcium is prescribed by 83.9% of the respondents. The majority of the interviewed sample (58.6%) requests uterine artery Doppler imaging to predict preeclampsia and 31.7% do not request any additional test. When the analysis was performed by region, only the northern region differed from the other Brazilian regions regarding the use of ASA and calcium for preeclampsia prevention. While more than 90% of physicians in the other regions prescribe ASA, 40% in the northern region do not use it (*p* < 0.0001). Regarding calcium, 30% of physicians in northern Brazil do not use the drug for preeclampsia prevention, a percentage that also differs from the other regions where the medication is prescribed by 80 to 90% of physicians (*p* = 0.021).

**Conclusions:**

The vast majority of Brazilian physicians prescribe low-dose aspirin and calcium carbonate to prevent preeclampsia in high-risk pregnant women. In addition to the identification of clinical risk factors, most doctors use Doppler of the uterine arteries as a predictive method. In the northern region of Brazil, physicians use aspirin and calcium less frequently for preventing preeclampsia compared to the rest of the country.

## Introduction

Preeclampsia (PE) is a multisystem disease that is characterized by new onset of hypertension and proteinuria, or hypertension and significant target-organ dysfunction with or without proteinuria, in the last half of pregnancy or postpartum. The genesis of the disease, which is multifactorial (genetic, immunological, environmental), is established in early pregnancy and is characterized anatomically by abnormal remodeling of maternal spiral arteries in the placenta (1).

Severe, but with a highly variable spectrum of severity, PE has important immediate and long-term impacts on a woman's future life. Preeclampsia affects up to 8% of all pregnancies worldwide (2) and is an important cause of maternal and perinatal morbidity and mortality. However, most adverse outcomes occur in low- and middle-income settings (2). For example, in Brazil where the maternal mortality ratio is about 56 deaths per 100,000 live births (and has remained stable over the last 10 years) (3), PE alone accounts for 20% of these deaths. In addition, 20% of all premature births in Brazil are related to PE and more than half are elective deliveries due to indications related to this condition (4, 5).

Considering the importance of PE, especially in Brazil where its prevalence and maternal mortality secondary to the disease are high, the screening of pregnant women at greater risk of developing this condition and the application of preventive measures are essential. However, the prediction of PE is a difficult task due to its complex etiology, knowledge gaps in its pathophysiology, diverse clinical presentations, and heterogeneity between populations.

Some studies have shown good predictive power for preterm PE of a model that combines clinical history (identification of risk factors), blood pressure measurement, Doppler imaging of the uterine arteries, and biomarkers (PAPP-A and PlGF) (6–8). Recently, the International Federation of Gynecology and Obstetrics (FIGO) issued its position on the role of combined screening in the universal screening for PE (9). However, this recommendation is still controversial and widely discussed in different countries. The National Specialized Commission (NSC) of Hypertension in Pregnancy of the Brazilian Federation of Gynecology and Obstetrics (FEBRASGO) published its position to reinforce local recommendations on PE prediction based only on the identification of risk factors and blood pressure measurement (10).

According to the NSC of Hypertension in Pregnancy, in agreement with some international guidelines such as those of the American College of Obstetricians and Gynecologists (ACOG) (11) and the US Preventive Services Task Force recommendations (12), evidence for the use of combined prediction models lacks external validation and implementation studies to demonstrate their predictive accuracy for clinical use. Thus, as also suggested by FIGO (for countries where combined screening does not appear to be cost effective) (9), these entities have recommended the prediction of PE based on the following risk factors: maternal age > 35 years, nulliparity, history of PE, interpregnancy interval < 12 or > 72 months, assisted reproduction, twin pregnancy, family history of PE (mother and sisters with PE), obesity, Afro-Caribbean descent, and presence of clinical conditions (chronic arterial hypertension, diabetes type 1 and 2, kidney disease, systemic lupus erythematosus, and antiphospholipid antibody syndrome) (9–14).

Recently, another biomarkers, like the soluble fms-like tyrosine kinase 1 (sFlt-1), endoglin, PlGF, neurokinin-B have been used to try to predict the preeclampsia's onset and/or preeclampsia's complications (15–20). The measurement of sFlt-1/PlGF ratio or PlGF alone is endorsed by some guidelines to rule out PE, since the negative predictive value of these tests are very high and reliable (14, 21). Neurokinin-B, a neuropeptide produced by the placenta, also can be a possible role in the pathophysiology of the disease. It is possible that neurokinin-B might act on peripheral neurokinin 3 receptors and neurokinin 1 receptors on platelets and monocytes, causing complications seen in late pre-eclampsia. However, the role of neurokinin-B as a predictive test is still debatable and requires clarification in the literature (19, 20).

In addition to the discussion on the best approach to prediction, the use of acetylsalicylic acid (ASA) for PE prevention has also been questioned not regarding its benefit and safety but regarding its correct administration (when to start and interrupt its administration and especially its dose). The current recommendation of FIGO (9) consists of the oral administration of ASA at a dose of 150 mg at night and calcium supplementation for women with inadequate intake (< 800 mg/day), emphasizing that this preventive measure should be based on positive screening. However, the FIGO recommendation of the ASA dose is fundamentally based on the ASPRE trial (8), which was not designed to compare the benefit of different ASA doses and always used the dose of 150 mg/day (8, 10).

In Brazil, 100-mg tablets are provided by the public health system. This concentration is already higher than the American recommendation, which is 81 mg/day (11, 12). Thus, the NSC of Hypertension in Pregnancy of FEBRASGO, in its recently published recommendation, maintains the aspirin dose of 100 mg/day (10). The drug should be started at 12 weeks of gestation (or, if necessary, from the beginning of prenatal care). Some authors propose that the benefits are achieved with the introduction of the drug until 16–20 weeks (10–14).

In view of this scenario of doubts and statements, we propose this national survey to identify the performance profile of Brazilian physicians involved in the care of pregnant women in the national territory regarding the prediction and prevention of PE in order to provide concrete evidence of their actions. These findings will contribute to developing strategies designed to improve medical practice in an attempt to reduce maternal and, consequently, neonatal morbidity and mortality in Brazil. The aims of this study were: (1) to evaluate the frequency of prescription of ASA and/or calcium for PE prevention by obstetricians in each region of Brazil; (2) to assess the frequency of use of PE prediction methods by the group of physicians studied, and (3) to describe the profile of Brazilian obstetricians who use PE prediction and prevention methods according to region and place of work.

## Methods

In this cross-sectional, descriptive study, a survey was developed using Google Forms and physicians who received it chose on their own free will to participate in the study. First, the participant signed the free and informed consent form, also accessed electronically, and then received the survey that consisted of 13 multiple-choice questions. It took about 5 min to complete the whole questionnaire, which was sent to obstetricians across Brazil by e-mail that contained an electronic link to the survey. The e-mails were sent by the secretariat of FEBRASGO and its federated members in Brazil to their mailing list members and the electronic link was also posted on different WhatsApp groups of obstetricians in Brazil.

The period for answering the survey was 6 months (1 October 2021 to 30 March 2022), when it was closed and the data were submitted to statistical analysis. The sample size was calculated a priori to obtain the maximum *n*. Assuming a prevalence of ASA prescription by obstetricians of 50%, an absolute error of 5% and a confidence coefficient of 95%, the inclusion of 385 sample units was estimated, according to the following formula (n = (z^2^ (1-*y*)/2*p*(1-*p*))/*d*^2^). The Excel program was used for tabulation and descriptive analysis of the data.

The study was linked to the University of São Paulo in Ribeirão Preto and was approved by the institutional Ethics Committee (Approval number 4.908.376).

## Results

**Figure 1:**
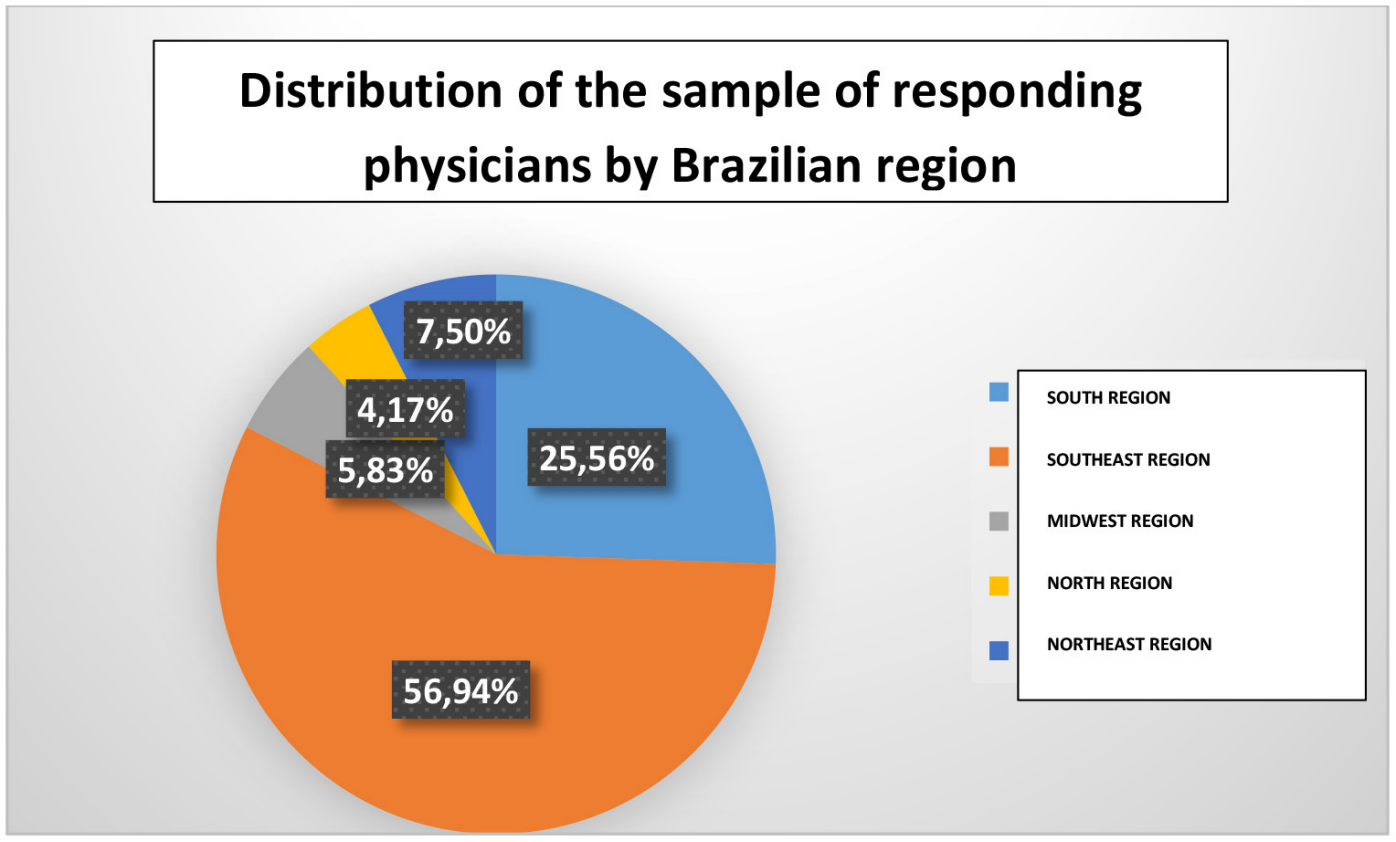
Distribution of the sample of responding physicians by Brazilian region.

The sample consisted of 360 respondents. Of these, 56.9% were from the southeast region and 25.5% from the south region (Figure 1). Obstetricians from the five different Brazilian regions participated in the survey (100% coverage). In addition, there were professionals from 23 (85%) of the 27 Brazilian states (including Distrito Federal). It is important to highlight that 71.1% of the respondents lived in cities with more than 200,000 inhabitants.

Most respondents (69.46%) work in a private office (not exclusively and 26% exclusively). Half of the interviewees (50.5%) work in a university service (not exclusively) and 40.2% work in a Basic Health Unit (UBS)/health center (not exclusively). Table 1 shows the range of professional activity of the respondents.

**Table 1:** Professional activity.

**What is the range of your professional activity?**	**n**	**%**
Private office	94	26.11
Private office, university service	73	20.28
University service	48	13.33
UBS/health center	37	10.28
UBS/health center, private office	47	13.06
UBS/health center, private office, university service	36	10
UBS/health center, university service	25	6.94

UBS, Basic Health Unit.

The vast majority of participants (94.72%) prescribe ASA for preeclampsia prevention, with 80.3% prescribing a dose of 100 mg/day. Table 2 shows the ASA doses prescribed by the responding physicians.

**Table 2:** Dose of acetylsalicylic acid prescribed for preeclampsia prevention.

**What ASA dose do you prescribe?**	**n**	**%**
100 mg/day	273	80.29
150 mg/day	56	16.47
50 mg/day	2	0.59
500 mg/day	1	0.29
75 mg/day	2	0.59
81 mg/day	6	1.76

ASA, acetylsalicylic acid.

**Figure 2:**
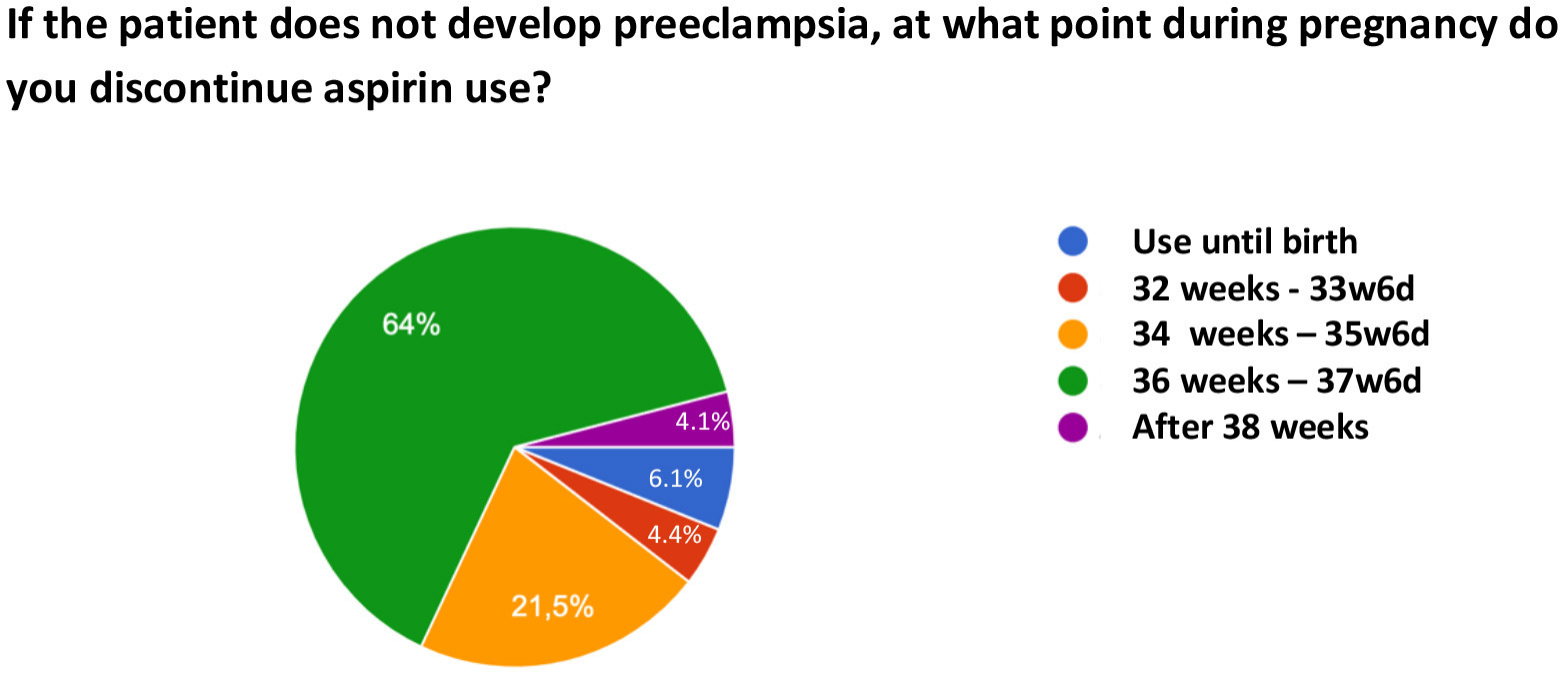
Time of discontinuation of acetylsalicylic acid use for preeclampsia prevention during pregnancy.

**Figure 3:**
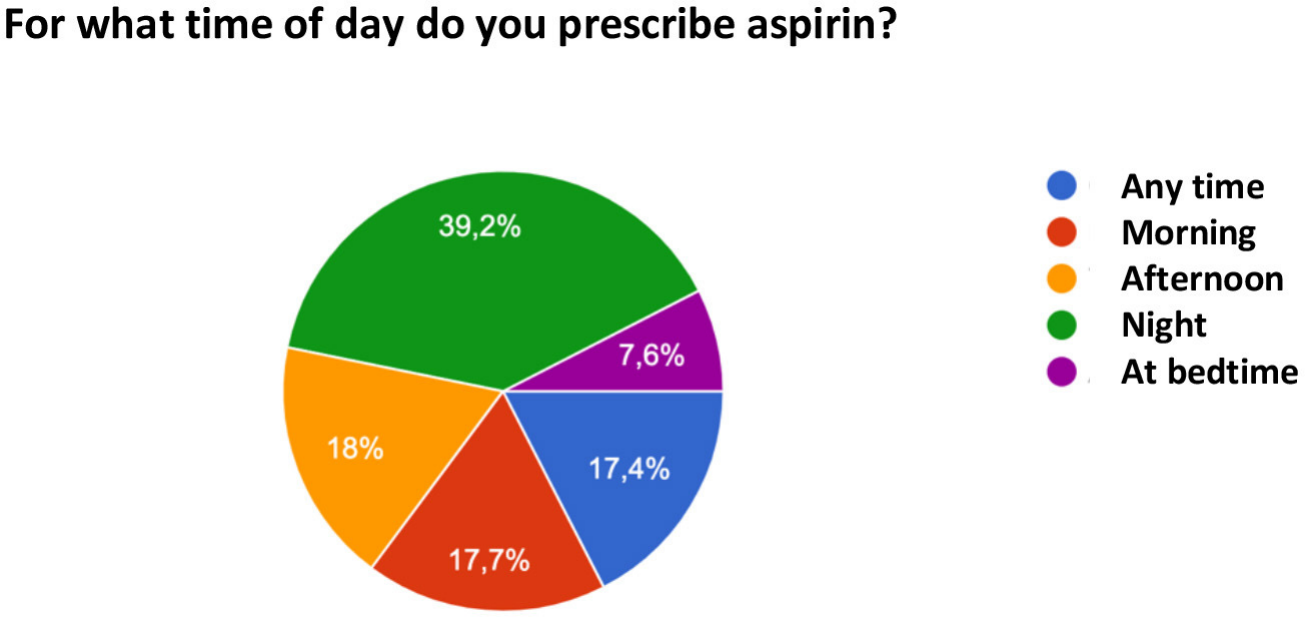
Time of day recommended by the respondents for acetylsalicylic acid use.

Regarding the initiation of ASA use, 70% of the sample ideally introduces the medication between 12–16 weeks and 16% responded that they ideally initiate it in the first trimester. With respect to the time of discontinuation of ASA, 64% of the respondents recommend its discontinuation between 36 and 38 weeks, 21.5% between 34 and 36 weeks, 4.4% between 32 and 34 weeks, 4.1% after 38 weeks, and 6.1% prescribe its use until birth. Figure 2 illustrates the time of discontinuation of ASA during pregnancy and Figure 3 shows the recommended time of day for medication use.

**Figure 4:**
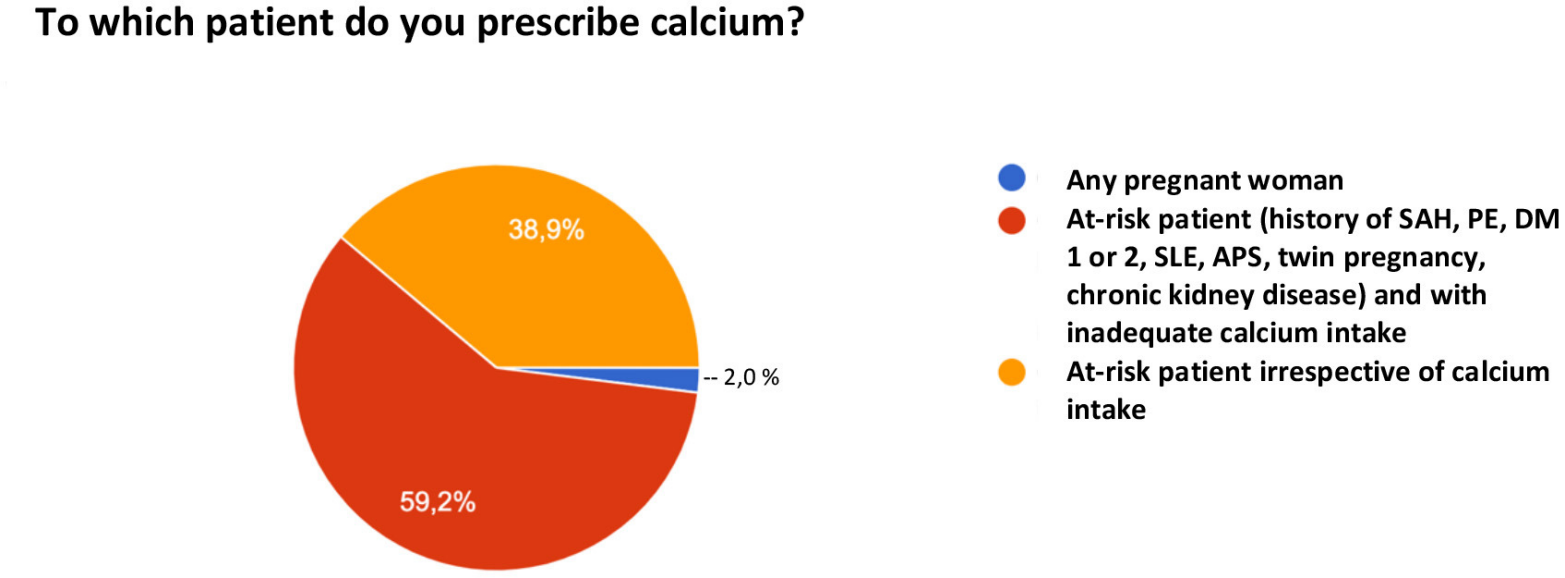
Use of calcium for preeclampsia prevention.

When asked about the use of calcium for PE prevention, 16.1% of the participants do not prescribe their patients calcium carbonate for this purpose. Among the 83.9% who do, 2% prescribe universal supplementation for any pregnant woman, 55% for pregnant women at risk who have inadequate intake, and 38.9% for any pregnant woman at risk irrespective of her intake (Figure 4).

**Figure 5:**
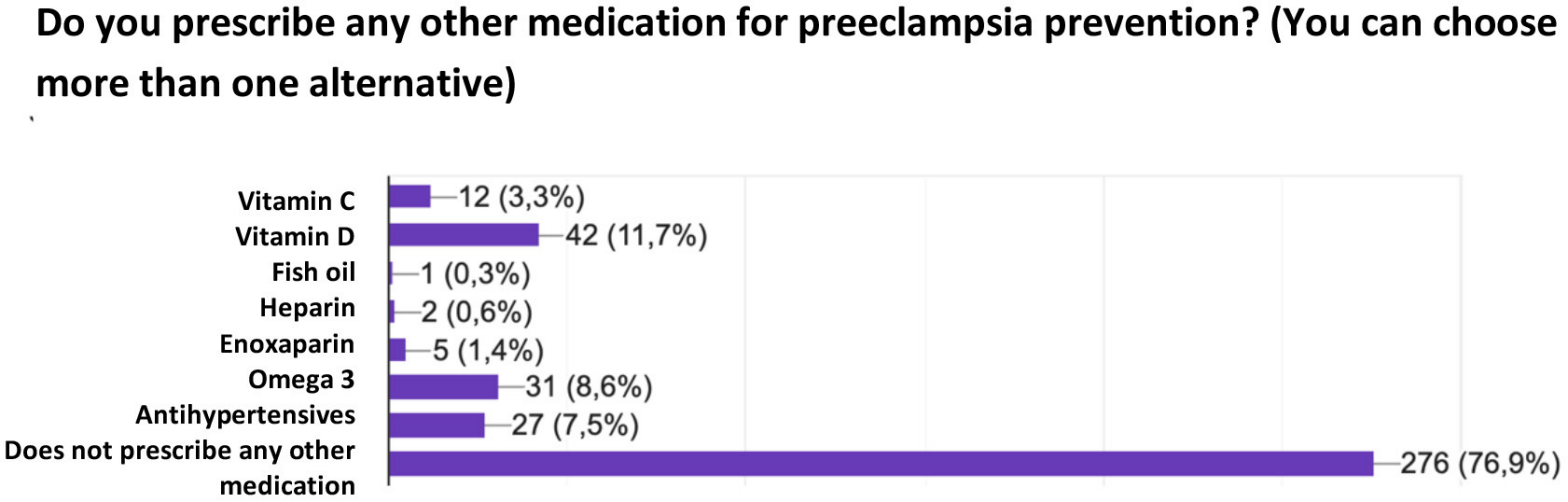
Medications other than acetylsalicylic acid and/or calcium prescribed by the respondents for preeclampsia prevention.

When asked about the use of any other drug for PE prevention, 76.9% of the sample answered that they do not use any additional drug other than ASA and/or calcium. Figure 5 shows the other medications prescribed by the population for PE prevention.

**Figure 6:**
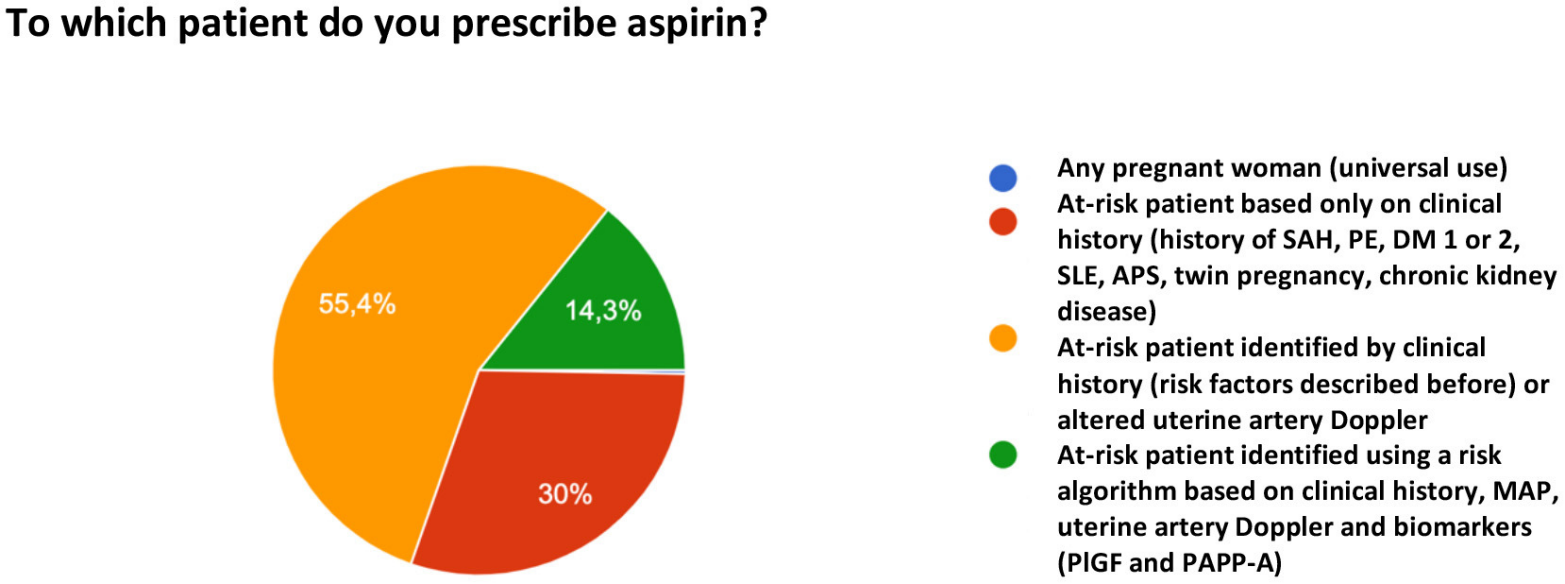
Reason for the indication of acetylsalicylic acid for preeclampsia prevention.

When asked to which patients to prescribe ASA for PE prophylaxis, 55.4% of the participants reported that they prescribe the drug based on risk factors identified in the clinical history and/or altered uterine artery Doppler velocimetry. Thirty percent prescribe ASA only based on the clinical history of risk factors and 14.3% reported the use a multifactorial algorithm (clinical history, mean arterial pressure [MAP], uterine artery Doppler, and biomarker measurement). One participant reported the universal use of ASA for PE prevention (Figure 6).

**Figure 7:**
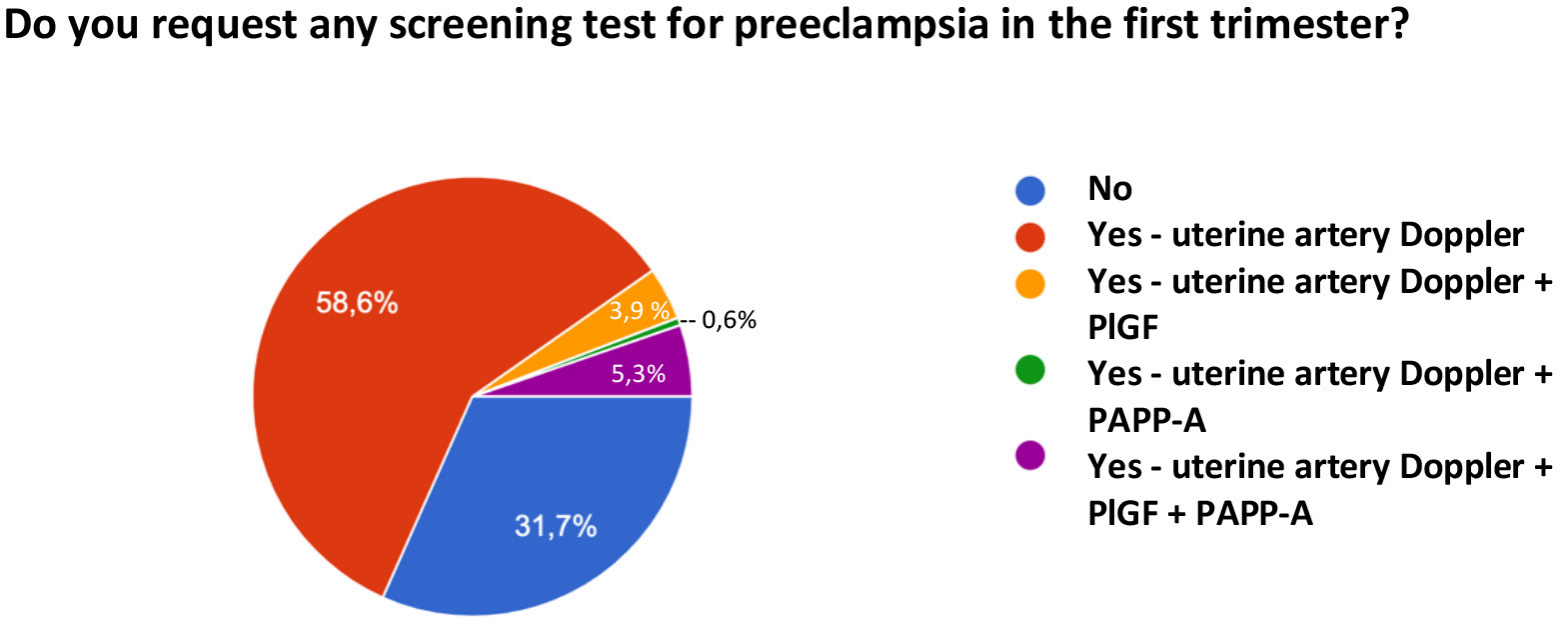
Tests requested for the prediction of preeclampsia by the population studied.

Regarding the prediction of preeclampsia, in addition to the identification of clinical risk factors, 31.7% of the sample does not request any other type of test, while 58.6% reported the use of uterine artery Doppler, 5.3% of uterine artery Doppler and biomarker measurement (PAPP-A and PlGF), 3.9% of Doppler and PlGF only, and 0.6% of Doppler and PAPP-A only. Figure 7 shows the methods for predicting PE used by the interviewed sample.

Finally, comparison of the use of ASA for PE prevention by Brazilian region revealed a significant difference for the northern region where 40% of respondents do not prescribe ASA, while the prescription of the drug exceeds 90% in all other regions (p < 0.0001). In contrast, the prescription of calcium exceeds 90% only in the Midwest region. In the southeast region, 88% of the respondents prescribe calcium, while this percentage does not reach 80% in the other three regions (south, north, and northeast). However, the greatest difference was observed in the northern region, where more than 30% of physicians do not use calcium (*p* = 0.021). Table 3 shows the comparison of prevention methods between Brazilian regions.

**Table 3:** Characterization of the sample according to region.

	**Region**					
	**Midwest**	**Northeast**	**North**	**Southeast**	**South**	***p*** -value^*^
**Do you prescribe aspirin for PE prevention?**						
No	0 (0)	2 (7.41)	6 (40)	5 (2.44)	6 (6.52)	<0.0001
Yes	21 (100)	25 (92.59)	9 (60)	200 (97.56)	86 (93.48)	
**To which patient do you prescribe ASA?**						
^*^At-risk patient based only on clinical history (history of SAH, PE, DM 1 or 2, SLE, APS, twin pregnancy, chronic kidney disease)	5 (23.81)	6 (23.08)	4 (36.36)	59 (29.8)	30 (34.48)	0.9487
^*^At-risk patient identified by clinical history (risk factors) or altered uterine artery Doppler	12 (57.14)	16 (61.54)	6 (54.55)	109 (55.05)	47 (54.02)	
^*^At-risk patient identified using a risk algorithm based on clinical history, MAP, uterine artery Doppler, and biomarkers (PAPP-A and PlGF)	4 (19.05)	4 (15.38)	1 (9.09)	30 (15.15)	10 (11.49)	
**Do you prescribe calcium for PE prevention?**						
No	1 (4.76)	6 (22.22)	4 (30.77)	24 (11.94)	22 (23.91)	0.021
Yes	20 (95.24)	21 (77.78)	9 (69.23)	177 (88.06)	70 (76.09)	
**To which patient do you prescribe calcium?**						
^*^At-risk patient (history of SAH, PE, DM 1 or 2, SLE, APS, twin pregnancy, chronic kidney disease) with inadequate calcium intake	9 (45)	15 (71.43)	6 (54.55)	109 (60.22)	42 (57.53)	0.2798
^*^At-risk patient irrespective of calcium intake	11 (55)	5 (23.81)	4 (36.36)	68 (37.57)	31 (42.47)	
^*^Any pregnant woman	0 (0)	1 (4.76)	1 (9.09)	4 (2.21)	0 (0)	
**Do you request any screening test for PE?**						
No	4 (19.05)	7 (25.93)	5 (33.33)	60 (29.27)	38 (41.3)	0.1775
Yes – uterine artery Doppler	17 (80.95)	17 (62.96)	9 (60)	120 (58.54)	48 (52.17)	
Sim - uterine artery Doppler + PlGF	0 (0)	3 (11.11)	1 (6.67)	25 (12.2)	6 (6.52)	

* Chi-square test.

## Discussion

This is the first survey on the prediction and prevention of PE that covers the whole Brazilian territory. Since PE is a serious disease and Brazil is an extensive country with very different cultures, a precarious public health system and protocols that often differ between public and supplementary health systems, mapping medical performance is essential to act locally where the greatest divergences exist in order to facilitate interventions.

Since there is no curative treatment other than childbirth, an intervention that can prevent PE is of extreme importance for maternal and child health worldwide, especially in lower-income countries where, in addition to a higher prevalence, the negative impact of the disease is far more devastating. Many different strategies designed to prevent PE have been investigated but most interventions are not successful, certainly because of the complex and multifactorial pathophysiology involved in the clinical syndrome (22). Prophylaxis with low-dose ASA is the most useful preventive pharmacological intervention, reducing the risk of new episodes of PE by 15%, the risk of perinatal mortality by 20%, and the risk of premature birth and intrauterine growth restriction by 20% (23).

Different guidelines recommend calcium supplementation for women with low intake of this mineral (24). Even in low-risk populations, a systematic review has demonstrated the benefit of calcium supplementation for reducing PE (25). In Brazil, the calcium intake of women of childbearing age is approximately 500 mg/day, which is below the currently recommended level (26). Thus, the NSC of Hypertension in Pregnancy of FEBRASGO recommends the administration of ASA and calcium supplementation to all pregnant women with clinical risk factors for PE as an important strategy for improving maternal and perinatal outcomes in the country, especially to those seen by the Brazilian public health system whose calcium intake may be lower (10).

Our study showed that the vast majority of physicians prescribe low-dose ASA for the prevention of PE (94.7%). Since almost 70% of the sample works in a private office and more than 40% in public health services (not exclusively in both cases), we believe that this preventive measure covers both patients from the Unified Health System (SUS) and from the supplementary health system. We did not assess patient adherence to the medication, which seems to be greater in the supplementary health system (access to medication, purchasing power, and education) (27), but the basis comes from the prescription and Brazilian colleagues are doing so.

Likewise, the prescribed ASA dose fully agrees with national and international recommendations. Most physicians in our sample (86%) prescribe a dose of 100 mg/day (which is the dose recommended by the NSC of Hypertension in Pregnancy of FEBRASGO and the tablet available at SUS) (10). If we consider international protocols that recommend doses between 75 and 150 mg/day, 99% of the physicians prescribe ASA within this dosage range (9–14).

Regarding calcium, its real prescription in Brazil for the prevention of PE is not as well established as that of ASA. Our study showed that, although most physicians use it, 16% of the sample does not prescribe the medication for this purpose. As previously mentioned, this is a missed window of opportunity given that calcium intake is generally inadequate in the Brazilian population (26).

The differences in the use of ASA and calcium carbonate between the different regions of Brazil must be highlighted. In the northern region, 40% of physicians do not prescribe ASA for PE prevention, while this rate exceeds 92% in all other regions. The same was observed for calcium, with 30% of respondents in this region not prescribing it. Considering its statistical significance (*p* < 0.0001 for ASA and p = 0.021 for calcium), this finding is important especially for guiding the bodies responsible for women's health in the country in order to design strategies aimed at reaching certain regions where prevention is failing. Due to the small *n* of participants in the northern region, the data need to be confirmed with new regional studies.

A matter of debate and lack of uniformity worldwide, the prediction of PE in the present Brazilian sample also shows heterogeneity. Only 31% of the respondents follow the local recommendations of the NSC of Hypertension in Pregnancy of FEBRASGO, i.e., predicting PE based only on risk factors observed in the clinical history and on blood pressure measurement (10). In addition to the identification of risk factor, 58% of the respondents use Doppler imaging of the uterine arteries and 5.3% reported the use of the complete algorithm, including Doppler and measurement of PlGF and PAPP-A in maternal blood, which is highly uncommon in Brazil since it requires the measurement of specific markers. It is important to highlight that, although it was not the aim of this study, combined prediction models have shown moderate predictive performance, with important limitations such as heterogeneity of the populations studied, low reproducibility of the methods used (mainly uterine artery Doppler), and a substantial lack of external validation. Evidence to support the use of these PE prediction models in clinical decision-making is limited and their predictive performance must be examined and validated locally before they can be considered for use in clinical practice (28).

As strengths of our study, we highlight the novelty and originality of a population survey on such an important topic for the health of Brazilian women and the fact that the study involved participants from all regions of the country. Certainly, the results obtained by mapping medical performance regarding the prediction and prevention of PE will serve as a basis for the development of local and specific measures and strategies aimed at improving healthcare practices.

Although we were able to include respondents from the five regions of Brazil and we reached a number very close to the calculated *n* (calculated sample size of 385 participants for a power of 95% and 360 were included), there was a higher percentage of participants from the southeast and south regions in our sample, a fact that may lead to random selection bias and may compromise data analysis, especially when the sample is divided by region. This is a limitation of the present study.

Brazilian people has a low intake of calcium, as we mentioned above (26). It is reasonable that it is one of the possible causes of the high prevalence of preeclampsia in Brazil. However, some papers have suggested that another substances, like vanadium, could have a possible relation with PE (29). We don't know the exposition level of Brazilians to vanadium or other minerals like lead or cadmium and we didn't do any mention about that.

Finally we have to comment the COVID-19 pandemic in the preeclampsia's scenario. Our study was conducted during the pandemic and many articles showed the impact of the COVID-19 in the obstetrics outcomes, with increase of maternal mortality and morbidity (30–32). Some papers revealed a strong association of PE and COVID-19, with increase of preeclampsia prevalence or associated morbidity and bad outcomes (32–36). So, all of actions that we can do to mitigate the onset or the complications of preeclampsia, like adequate prediction and prevention, are welcome, particularly in the pandemic period, where the impact of the disease was so powerful.

The challenge for Brazilian health does not only comprise medical but also political and social issues and requires constant articulation between governments and society. Furthermore, in a country with the size of Brazil with sociocultural and financial differences, frequent mapping of the performance of health professionals, such as done in the present study, is extremely valuable to identify possible opportunities for action and improvement.

## Conclusion

The vast majority of Brazilian physicians prescribe low-dose ASA and calcium carbonate for PE prevention among at-risk pregnant women. In addition to identifying clinical risk factors, most respondents use Doppler imaging of the uterine arteries as a predictive method. The percentages of ASA and calcium prescription for PE prevention are lower in the northern region when compared to the other Brazilian regions.

## Data availability statement

The raw data supporting the conclusions of this article will be made available by the authors, without undue reservation.

## Author contributions

All authors listed have made a substantial, direct, and intellectual contribution to the work and approved it for publication.

## Conflict of interest

The authors declare that the research was conducted in the absence of any commercial or financial relationships that could be construed as a potential conflict of interest.

## Publisher's note

All claims expressed in this article are solely those of the authors and do not necessarily represent those of their affiliated organizations, or those of the publisher, the editors and the reviewers. Any product that may be evaluated in this article, or claim that may be made by its manufacturer, is not guaranteed or endorsed by the publisher.

## References

[1] Magge LA Brown MA Hall DR Gupte S Hennessy A Karumanchi SA. The 2021 International Society for the Study of Hypertension in Pregnancy classification, diagnosis & management recommendations for international practice. Pregnancy Hypertens. (2022) 27:148–69. 10.1016/j.preghy.2021.09.008 35066406

[2] Duley L. The global impact of pre-eclampsia and eclampsia. Semin Perinatol. (2009) 33:130–7. 10.1053/j.semperi.2009.02.010 19464502

[3] Ministério da Saúde. Secretaria de Vigilância em Saúde. Departamento de Análise de Saude e Vigilância de Doenças Não Transmissíveis. Painel de monitoramento da mortalidade materna. (2019). Available online at: http://svs.aids.gov.br/dantps/centrais-de-conteudos/paineis-de-monitora-mento/mortalidade/materna/ (accessed December 10, 2019).

[4] Passini R Jr Tedesco RP Marba ST Cecatti JG Guinsburg R Martinez FE. Brazilian network of studies on reproductive and perinatal health. Brazilian multicenter study on prevalence of preterm birth and associated factors. BMC Preg Childbirth. (2010) 10:22. 10.1186/1471-2393-10-22 20482822 PMC2889846

[5] Souza RT Cecatti JG Passini R Jr Tedesco RP Lajos GJ Nomura ML. Brazilian multicenter study on preterm birth study group. The burden of provider-initiated preterm birth and associated factors: evidence from the Brazilian Multicenter Study on Preterm Birth (EMIP). PLoS ONE. (2016) 11:e0148244. 10.1371/journal.pone.0148244 26849228 PMC4743970

[6] Akolekar R Syngelaki A Poon L Wright D Nicolaides KH. Competing risks model in early screening for preeclampsia by biophysical and biochemical markers. Fetal Diagn Ther. (2013) 33:8–15. 10.1159/000341264 22906914

[7] O'Gorman N Wright D Syngelaki A Akolekar R Wright A Poon LC. Competing risks model in screening for pre-eclampsia by maternal factors and biomarkers at 11-13 weeks gestation. Am J Obstet Gynecol. (2016) 214:103.e1–103.e12. 10.1016/j.ajog.2015.08.034 26297382

[8] Rolnik DL Wright D Poon LCY Syngelaki A O'Gorman N Paco Matallana C. ASPRE trial: performance of screening for preterm pre-eclampsia. Ultrasound Obstet Gynecol. (2017) 50:492–5. 10.1002/uog.18816 28741785

[9] Poon LC Shennan A Hyett JA Kapur A Hadar E Divakar H. The International Federation of Gynecology and Obstetrics (FIGO) initiative on pre-eclampsia: a pragmatic guide for first-trimester screening and prevention. Int J Gynaecol Obstet. (2019) 145:1–33. 10.1002/ijgo.12802 31111484 PMC6944283

[10] De Oliveira LG Diniz ALD Prado CAC Cunha Filho EV Souza FLP Korkes HA. Pre-eclampsia: Universal Screening or Universal Prevention for Low and Middle-Income Settings? Statement of the National specialized commission of hypertension in pregnancy of the Brazilian association of gynecology and obstetrics federation – FEBRASGO. Rev Bras Ginecol Obstet. (2021) 43:61–5. 10.1055/s-0040-1713803 33513638 PMC10183869

[11] Gestational hypertension and preeclampsia. ACOG Practice Bulletin No. 222. American College of obstetricians and gynecologists. Obstet Gynecol. (2020) 135:e237–60. 10.1097/AOG.0000000000003891 32443079

[12] Grossman DC Curry SJ. Barry MJ Davidson KW Doubeni CA. Screening for preeclampsia: US preventive services task force recommendation statement. JAMA. (2017) 317:1661. 10.1001/jama.2017.3439 28444286

[13] Meher S Duley L Hunter K Askie L. Antiplatelet therapy before or after 16 weeks' gestation for preventing preeclampsia: an individual participant data meta-analysis. Am J Obstet Gynecol. (2017) 216:121–128.e2. 10.1016/j.ajog.2016.10.016 27810551

[14] NICE guideline. NICE guideline 133; Hypertension in pregnancy: diagnosis and management. Available online at: www.nice.org.uk/guidance/ng133 (accessed June 25, 2019).

[15] Hurrell A Beardmore-Gray A Duhig K Webster L Chappell LC Shennan AH. Placental growth factor in suspected preterm pre- eclampsia: a review of the evidence and practicalities of imple- mentation. BJOG. (2020) 127:1590–7. 10.1111/1471-0528.16425 32701207

[16] Zeisler H Llurba E Chantraine F Vatish M Staff AC Sennström M. Predictive value of the sFlt-1: PlGF ratio in women with suspected preeclampsia. N Engl J Med. (2016) 374:13–22. 10.1056/NEJMoa1414838 26735990

[17] Cerdeira AS O'Sullivan J Ohuma EO Harrington D Szafranski P Black R. Randomized Interventional study on prediction of preeclampsia/ eclampsia in women with suspected preeclampsia INSPIRE. Hypertension. (2019) 74:983–90. 10.1161/HYPERTENSIONAHA.119.12739 31401877 PMC6756298

[18] Duhig KE Myers J Seed PT Sparkes J Lowe J Hunter RM. on behalf of the PARROT trial group. Placental growth factor testing to assess women with suspected pre-eclampsia: a multicentre, pragmatic, stepped-wedge cluster-randomised controlled trial. Lancet. (2019) 393:1807–18. 10.1016/S0140-6736(18)33212-4 30948284 PMC6497988

[19] Salman H Shah M Ali A Aziz A Vitale SG. Assessment of relationship of serum neurokinin-b level in the pathophysiology of pre-eclampsia: a case–control study. Adv Ther. (2018) 35:1114–21. 10.1007/s12325-018-0723-z 29923045

[20] Page NM Woods RJ. Gardiner SM Lomthaisong K Gladwell RT Butlin DJ. Excessive placental secretion of neurokinin B during the third trimester causes pre-eclampsia. Nature. (2000) 405:797–800. 10.1038/35015579 10866201

[21] Costa ML Cavalli RC Korkes HA Cunha Filho EV Peraçoli JC. Diagnosis and management of preeclampsia: suggested guidance on the use of biomarkers. Rev Bras Ginecol Obstet. (2022). 10.1055/s-0042-1744286 35468644 PMC9948147

[22] August P Jeyabalan A. Preeclampsia: Prevention. (2022). Available online at: https://www.uptodate.com/contents/preeclampsia-prevention?search=preeclampsia%20prevention&source=search_result&selectedTitle=1 150&usage_type=default&display_rank=1

[23] HendersonJTVescoKKSengerCAThomasRGRedmondN. Aspirin use to prevent preeclampsia and related morbidity and mortality: updated evidence report and systematic review for the US preventive services task force. JAMA. (2021) 326:1192. 10.1001/jama.2021.855134581730

[24] World Health Organization. WHO Recommendations for Prevention and Treatment of Pre-Eclampsia and Eclampsia. Geneva: WHO. (2011). 23741776

[25] Sun X Li H He X Li M Yan P Xun Y. The association between calcium supplement and preeclampsia and gestational hypertension: a systematic review and meta-analysis of randomized trials. Hypertens Preg. (2019) 38:129–39. 10.1080/10641955.2019.1593445 30935246

[26] Camargo EB Moraes LF Souza CM Akutsu R Barreto JM Silva EMK. Survey of calcium supplementation to prevent preeclampsia: the gap between evidence and practice in Brazil. BMC Pregnancy Childbirth. (2013) 13:206. 10.1186/1471-2393-13-206 24215470 PMC3832745

[27] Tavares NUL Bertoldi AD Mengue SS Arrais PSD Luiza VL Oliveira MA. Fatores associados à baixa adesão ao tratamento farmacológico de doenças crônicas no Brasil. Rev Saude Publica. (2016) 50:10s.27982378

[28] Snell KIE Allotey J Smuk M Hooper R Chan C Ahmed A. External validation of prognostic models predicting pre-eclampsia: individual participant data meta-analysis. BMC Med. (2020) 18:302. 10.1186/s12916-020-01766-9 33131506 PMC7604970

[29] Ovayolu A Turksoy VA Gun I Karaman E Dogan I Turgut A. Analyses of maternal plasma cadmium, lead, and vanadium levels in the diagnosis and severity of late-onset preeclampsia: a prospective and comparative study. J Matern Fetal Neonatal Med. 35:4803–9 10.1080/14767058.2020.1864318 33406955

[30] Souza RT Cecatti JG Pagagnella RC Ribeiro-Do-Valle CC Luz AG Lajos GJ. The COVID-19 pandemic in Brazilian pregnant and postpartum women: results from the REBRACO prospective cohort study. Sci Rep. (2022) 12:11758. 10.1038/s41598-022-15647-z 35817818 PMC9272878

[31] La Verde M Riemma G Torella M Cianci S Savoia F Licciardi F. Maternal death related to COVID-19: a systematic review and meta-analysis focused on maternal co-morbidities and clinical characteristics. Int J Gynecol Obstet. (2021). 154:212– 219. 10.1002/ijgo.13726 33930185 PMC9087672

[32] Jamieson DJ Rasmussen SA. An update on COVID-19 and pregnancy. Am J Obstet Gynecol. (2022) 226:177–86. 10.1016/j.ajog.2021.08.054 34534497 PMC8438995

[33] Papageorghiou AT Deruelle P Gunier RB Rauch S García-May PK Mhatre M. Preeclampsia and COVID-19: results from the INTERCOVID prospective longitudinal study. Am J Obstet Gyneco. (2021) 225:289.e1–17. 10.1016/j.ajog.2021.05.014 34187688 PMC8233533

[34] Conde-Agudelo A Romero R. SARS-CoV-2 infection during pregnancy and risk of preeclampsia: a systematic review and meta-analysis. Am J Obstet Gynecol. (2022) 226:68–89.e3. 10.1016/j.ajog.2021.07.009 34302772 PMC8294655

[35] Guida JP Cecatti JG Souza RT Pacagnella RC Ribeiro-Do-Valle CC Luz AG. Preeclampsia among women with COVID-19 during pregnancy and its impact on maternal and perinatal outcomes: results from a national multicenter study on COVID in Brazil, the REBRACO initiative. Preg Hypertens. (2022) 28:168–73. 10.1016/j.preghy.2022.05.005 35568019 PMC9085347

[36] Mendoza M Garcia-Ruiz I Maiz N Rodo C Garcia-Manau P Serrano B. Pre-eclampsia-like syndrome induced by severe COVID-19: a prospective observational study. BJOG. (2020) 127:1374–80. 10.1111/1471-0528.16339 32479682 PMC7300912

